# The Influence of Plant Material Enzymatic Hydrolysis and Extraction Conditions on the Polyphenolic Profiles and Antioxidant Activity of Extracts: A Green and Efficient Approach

**DOI:** 10.3390/molecules25092074

**Published:** 2020-04-29

**Authors:** Aneta Krakowska-Sieprawska, Katarzyna Rafińska, Justyna Walczak-Skierska, Bogusław Buszewski

**Affiliations:** 1 Department of Environmental Chemistry and Bioanalytics, Faculty of Chemistry, Nicolaus Copernicus University, Gagarina 7 St, PL-87-100 Torun, Poland; akra@doktorant.umk.pl (A.K.-S.); katraf@umk.pl (K.R.); 2 Interdisciplinary Centre of Modern Technologies, Nicolaus Copernicus University, Wilenska 4, 87-100 Torun, Poland; walczak-justyna@wp.pl

**Keywords:** enzyme assisted-supercritical fluid extraction, phenolic compounds, flavonoids, antioxidant activity, high performance liquid chromatography

## Abstract

The aim of this study was to develop a new comprehensive extraction protocol based on green technology for the enhanced release of polyphenolic compounds from plant cells. In this work, extracts from yerba mate and yellow lupine seed were obtained by using three different extraction techniques: maceration, supercritical fluid extraction with co-solvent (SFE) and enzyme assisted-supercritical fluid extraction with co-solvent (EA-SFE). Several experimental parameters such as time, type of solvent and co-solvent as well as CO_2_ flow rate were selected to obtain the highest extraction efficiency. The chemical profiles in the obtained extracts and their biological activity were evaluated. HPLC-MS/MS analysis indicated that the level of phenolic compounds in extracts from yerba mate obtained by EA-SFE was approximately five times higher than for maceration and 3.2 times higher than for SFE. In the case of extracts from yellow lupine seed an approximately 5.6-fold increase was observed in comparison with maceration and SFE with 96% MeOH, and 2.9 times for SFE with 96% EtOH. The developed protocol with a mix of enzymes commonly applied in the agricultural industry significantly raises the efficiency of liberation of secondary metabolites.

## 1. Introduction

Plants are basically an unlimited source of biologically active compounds, and the products obtained from them are increasingly being used not only in the pharmaceutical and cosmetics industries, but also in the food industry. The largest group of bioactive compounds in plants are polyphenols which exhibit antioxidant and antibacterial properties and can therefore be used as natural preservatives of cosmetic and food products. They can enhance their microbiological safety and extend the shelf-life. Moreover, the development of the organic cosmetic and food industry will provide in the future to much greater use of natural preservatives of plant origin but importantly they should also be obtained in the environmentally friendly way [1].

Yerba mate is a tea infusion made from the leaves of the tree *Ilex paraguariensis* A.St.-Hil., known and prepared by the Guarani tribe in the Parana basin, in order to reduce fatigue, drowsiness, lighten the mind and relieve hunger [2]. The most important compounds determining the properties of yerba mate are primarily polyphenolic compounds and purine alkaloids. It has been shown that their level in infusions is higher than in green and black tea. The most abundant polyphenols include caffeic acid and its derivatives, chlorogenic acid, kaempferol, quercitin, quercitinic acid, rutin, quinic acid, and gallic acid. In turn, the main purine alkaloids are caffeine (0.4–1.8%) and theobromine (0.15–0.8%). Moreover, this beverage is a rich source of mineral salts, containing potassium, magnesium and manganese. The content of bioactive compounds determines the antioxidant, anti-inflammatory, anti-bacterial, anti-cancer properties of this species of plant [3,4]. The substances mainly responsible for the specific taste of yerba mate are bitter, water-soluble saponins. A 1.2% saponin content was found in the holly leaves. Due to the fact that yerba mate displays a high antioxidant potential and a large content of bioactive compounds, it is a good material for isolation these compounds for further use in dietary supplements or as ingredients in formulated foods. [4].

*Lupinus luteus* L., known as yellow-lupine, is an annual plant species from the *Fabaceae* L. family. Due to its low content of harmful alkaloids is also called sweet edible lupine [5]. In Poland, yellow lupine is primarily used as a garden plant, and less often as feed for farm animals, while in Western Europe it is gaining more and more importance as an edible plant in the human diet. It is often used as a cooking grain, for the production of lupine flour and a great gluten-free supplement in the diet of diabetics or people in periods of convalescence [6]. Yellow lupine seeds are a rich source of protein (38–50%) and fat (5–20%). In addition, they contain a wide range of amino acids, especially arginine, as well as antioxidants (polyphenols). It is also worth mentioning that the high fiber content as well as the low glycemic index (used to assess blood glucose after ingestion) are an additional advantage in the culinary use of the plant. Our previous research showed that SFE extract from germinated yellow lupine has antibacterial properties without showing cytotoxicity [5,6].

Extraction is a key step in the isolation of biologically active compounds from plant materials and an appropriate selection of conditions, including an appropriate technique and solvent, allows one to get products with high activity. The variety of extraction techniques available in sample−extractant systems allows one to successfully select the one that will permit the isolation of selected compounds from the sample with the assumed yield and then determine them using a proper analytical technique. However, the key issue of the extraction process is the selection of an appropriate solvent. The type of solvent determines the isolation efficiency during the extraction process, and the highest efficiency can be obtained at the moment when the chosen solvent interacts specifically with the extracted compounds. The final selection of a given extraction technique should be additionally guided by “green chemistry” reasons, i.e., striving to use as little reagents as possible and avoiding the use of toxic reagents. Therefore, in comparison with conventional extraction techniques (classical solvent extraction/maceration, Soxhlet extraction, etc.), the preferred techniques are accelerated solvent extraction (ASE), ultrasound assisted extraction (ultrasound−assisted extraction or UAE), or supercritical fluid extraction (SFE) [7].

Due to the increasingly restrictive regulations regarding environmental protection and contemporary trends defined by the so-called “green chemistry principles” supercritical fluid extraction (SFE) has gained special interest among extraction techniques. The “ideal” extraction should be characterized by the kinetic, high yield and quantitative recovery of the extracted substances, which should not be degraded during the process, and the solvent used should be readily separated from the solute. The classical extraction techniques used to date in the isolation of organic compounds are very often time-consuming and require the use of a large amount of toxic solvents such as dichloromethane or methanol. Therefore, techniques that are close to the “ideal” are dynamically developed to the greatest possible extent. On the wave of this development, supercritical fluid extraction has become an interesting alternative to classical techniques, especially in the case of plant materials. In addition, the growing interest in natural products and the fact that they are characterized by low concentrations make supercritical fluid extraction a leading extraction technique when working with these products [8,9].

The mechanism which determines the extraction efficiency is the mass transport of the substance extracted from the interior of the plant material and then from the contact surface of the phases to the solvent. An important role in the extraction process is the mass transfer resistance associated with the structure of the raw material and the specific location of the extracted compounds. It is currently believed that bioactive compounds are bound to the cell wall polymers by many weak interactions like hydrogen bonds or hydrophilic interactions and therefore it is difficult to leach them out using conventional extraction methods. The presence of polysaccharides such as cellulose, pectin and hemicellulose reduces the extraction efficiency of conventional extraction methods.

An interesting approach that allows the release of the analyzed compounds is the use of enzymes for degrading the cell walls of plants. The destruction of cell wall integrity by the use of different enzymes aims to increase the isolation of bioactive plant components and thus improve the extraction efficiency. Enzymes, such as cellulase, β-glucosidases and pectinases are used for the hydrolysis and degradation of plant cell wall components. The effectiveness of enzymatic processes depends largely on the type of enzyme or enzyme mixture used and the selection of optimal conditions for their operation (pH, ionic strength, temperature). Therefore, the development of a new effective and selective protocols for increased recovery of bioactive substances, especially from plants in which the cell wall may be a physical barrier preventing effective extraction would be beneficial [9,10,11,12,13]. To date, there is little information about extraction of polyphenolic compounds by supercritical fluid extraction supported by enzymes from yerba mate and yellow lupine−two important species from a pharmaceutical and supplement industry point of view.

Based on the above considerations, the aim of this study was to develop a new method for isolation of secondary metabolites from plant material, with the final goal of enhancing the release of these bioactive compounds from plant cells. In the first step of our research, we focused on the selection of conditions for the isolation of biologically active compounds from the plant matrix by maceration (variable such as solvent) and supercritical fluid extraction using variables such as extraction time, type and flow of co-solvent, to increase the efficiency of the extraction process. In the next step of the study, we have proposed the use of a combination of enzymes that hydrolyze the plant cell walls. The studied mixture of enzymes such as cellulase, β-glucosidase or pectinase called Kemzyme^®^ was used to develop the protocol for isolation and determination of selected plant secondary metabolites. Our research offers a new EA-SFE method as a “green” protocol for the extraction of high-values phenolics and other antioxidants from yerba mate and yellow lupine, which could be used in the food, cosmetic and pharmaceutical industries.

## 2. Results and Discussion

### 2.1. Yields of Extractions

Yield is a first parameter which allows one to evaluate the effectiveness of an extraction process. In our work we have used the most popular solvents to evaluate their effectiveness in extraction of biological materials of different structures, i.e., seeds rich in proteins and vegetative tissues whose cell walls are rich in cellulose and pectin. Table 1 shows the percentage yields obtained by different extraction conditions, according to the procedure performed to obtain the extracts for each of the plants.

In conventional extraction methods, the decisive feature of the extraction efficiency is the polarity of the solvent. In the case of isolation of biologically active compounds, with increasing polarity of the solvent used, the efficiency of this process was expected to increase (Y_70% MeOH_ > Y_70% EtOH_ > Y_96% MeOH_ > Y_96% EtOH_) [14].

These assumptions did not work for tested plant materials, as we can see that differences between solvents are insignificant. For yellow lupine the highest yield was obtained for 96% methanol which can suggest that at least some of the extracted compounds show low polarity (Table 1).

For supercritical fluid extraction, values of yields were lower than in the case of maceration. For all tested plant extracts, the highest efficiency for SFE was obtained using 96% ethanol as a co-solvent. In the case of yerba mate extracts obtained by SFE, similar yield values are noticed in pairs of co-solvents: 96% ethanol–96% methanol (3.45–3.12%) and 70% ethanol–70% methanol (1.45–1.50%) (Table 1).

**Figure 1:**
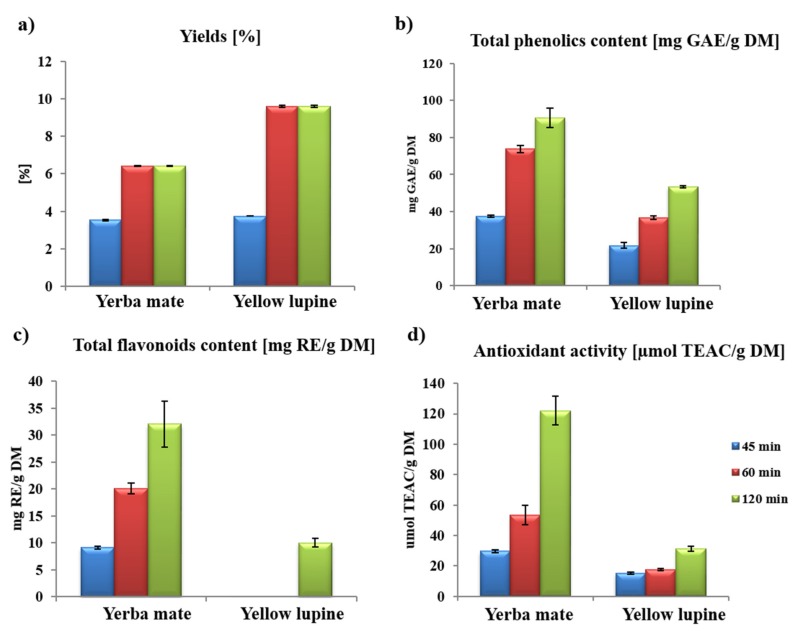
Yields (%) (**a**), total phenolic content (mg GAE/g DM) (**b**), total flavonoid content (mg RE/g DM) (**c**) and antioxidant activity obtained by the DPPH method (µmol TEAC/g DM) (**d**) of the extracts obtained by SFE with different duration of the process for different plants (T—50 °C, p—300 bar, flow rate CO_2_—4 mL/min, flow rate co-solvent (96% EtOH)—1 mL/min).

Determination of the yields of individual extraction variants differing in the duration of the process allows to see the dependence of the proportionality of extraction duration on its efficiency. In the case of extracts obtained with SFE, the highest levels of yield were obtained at 60 and 120 min of the process (Figure 1a) and reached 6.42% for yerba mate extracts and 9.60% for yellow lupine, which is twice as high as in the case of 45 min extraction. Literature data shows that with the increase of the dynamic extraction time when using constant pressure and temperature, an increase in the efficiency of the whole process is observed, and thus an increase in the amount of extracted compounds, especially those of high mass is expected [14].

Table 1 shows the efficiency obtained for SFE with different flow rate of co-solvent (96% EtOH). Based on one of the main principles of green chemistry to reduce the use of solvents in chemical processes, two SFE variants were compared with 96% ethanol as a co-solvent and the duration of a 120 min process for the two plants tested. As shown by the studies (Table 1) in the case of plant extracts obtained by SFE, we observed that the reduction of the solvent flow results in the increased extraction efficiency. The optimal solvent flow rate provides proper mas gradient between the solute in extracted plant material and solute already dissolved in solvent. In our study, a flow 0.4 mL/min maintained adequate solubility of the isolated compounds.

### 2.2. Total Content of Phenolic Compounds

In Table 1 the total phenolic content for the plant extracts obtained by the different techniques of extraction is shown. Extracts from yerba mate macerated with 96% ethanol had the highest content of phenolic compounds—135.48 mg GAE/g DM—which turned out to be the highest value among all plant macerates tested. The least effective solvent was 70% ethanol. Macerates from yellow lupine were characterized as the least rich in phenolic compounds in comparison with the other plant (Table 1). SFE extracts were much poorer in phenolic compounds. Only SFE extracts from yerba mate presented a relatively high content of phenolic compounds –51.95 mg GAE/g DM.

Obtained results have shown that ethanol extracts phenolic compounds much more efficiently from tested matrices than methanol. The lowest level of these compounds was obtained with using 70% methanol—15.22 mg GAE/g DM for extracts of yerba mate (Table 1). Extracts of yellow lupine in all four variants of the solvents were characterized by the lowest level of phenolic compounds among the tested matrices. Similar research on phenolic compounds in limnophile extracts (*Limnophila aromatica*) indicates that, as in Table 1, the efficiency of phenolic compounds extraction increases with the purity of ethanol, however, the highest level of these compounds was determined in methanol extracts [15]. For both tested plant materials, the highest content of phenolic compounds was present in extracts after a 120 min. SFE, i.e., 90.65 mg GAE/g DM for yerba mate extract and 53.45 mg GAE/g DM in the yellow lupine extract (Figure 1b).

The results obtained for supercritical extractions with different flow rate of co-solvent were at a similar level in both variants (Table 1). Besides that, only in extract from yerba mate macerated with 96% ethanol, higher content of polyphenolic compounds than in extracts obtained by SFE was observed. Studies on chamomile (*M. chamomilla*) [16,17] show results similar to those we have observed for yerba mate, whereby maceration is a significantly more effective method for the isolation of polyphenolic compounds from plant material than SFE. However, for yellow lupine it was the reverse, and SFE was much more efficient technique for obtaining polyphenolics than maceration with 96% (Table 1) which indicates a higher proportion of polyphenolic compounds with less polarity due to lower polarity of combination of scCO_2_ and EtOH.

### 2.3. The Total Content of Flavonoids

The total content of flavonoids in extracts from the different plants is presented in Table 1. As it can be seen for supercritical extracts, the highest level of flavonoids in the case of yerba mate was extracted using 96% ethanol as co-solvent—15.77 mg RE/g DM—while the lowest—6.32 mg RE/g DM—was obtained using 70% ethanol. In the yellow lupine extracts, in each of the four variants of the used co-solvent, the flavonoids were not extracted at the level which can be determined by the method used. As we have shown in the research (Table 1), supercritical fluid extraction enables the isolation of polar compounds such as phenolics and flavonoids from plant material. Although the scCO_2_ as a main solvent in this case is non-polar, the addition of a polar co-solvent (EtOH, MeOH) allows the spectrum of isolated compounds to be extended to include more polar substances.

Like for the total content of phenolic compounds, the highest values of flavonoids were achieved for extracts obtained during 120 min of extraction process, i.e., 32.05 mg RE/g DM for yerba mate and 10.05 mg RE/g DM for yellow lupine (Figure 1c). It is worth noting that in the case of yellow lupine extracts, the flavonoid content was found only in the extraction variant with time of 120 min.

In the case of maceration similar as for *Lepidium sativum*, 96% ethanol was the most efficient solvent for flavonoids isolation (Table 1) [18]. For yerba mate extract the level of flavonoids was 21.39 mg RE/g DM and for yellow lupine extract, 5.55 mg RE/g DM, respectively (Figure 1c).

### 2.4. Antioxidant Activity

The antioxidant properties of tested extracts were measured according to the DPPH method. The obtained results are shown in Table 1. In the case of SFE, the highest total antioxidant capacity was noticed for extracts obtained using 96% ethanol as a co-solvent. Literature data indicate a relationship between the content of polyphenolic compounds and antioxidant potential [19,20], resulting from their structure and the possibility of neutralization of free radicals by the formation of stabilized phenoxy radicals. Our research seems to confirm this correlation. Ethanolic extracts (96%) characterized by the highest level of polyphenolic compounds showed the highest antioxidant potential determined by the DPPH method (Table 1). However, it should be noted that antioxidant properties are also influenced by other classes of compounds that may be present (eg. saponins, carotenoids, some vitamins) [21].

The correlation between the content of phenolic compounds and the antioxidant potential measured by the DPPH method again seems to confirm that the higher the level of these compounds in the plant extract, the higher this potential. Extracts obtained by a 120 min extraction process were characterized by a higher level of polyphenolic compounds than those obtained after a shorter time, also showed correspondingly higher antioxidant potentials (Figure 1b,d). In this variant the antioxidant activity for yerba mate extract was 122.24 mg TEAC/g DM and for yellow lupine extract 31.34 mg TEAC/g DM. Moreover, for yerba mate, which is attributed antioxidant properties, extracts obtained by SFE with 96% EtOH as a co-solvent were characterized by the highest level of antioxidant potential measured by DPPH. Higher solvent flows were more efficient in isolation of antioxidant compounds, as for total phenolic content (Table 1).

In case of the maceration, it is impossible to clearly indicate a correlation between the content of polyphenolic compounds and the antioxidant potential measured by the DPPH method (Table 1). This fact suggests that, through maceration, other unidentified compounds that affected this value were probably isolated. Our results showed that supercritical fluid extraction is a promising method of obtaining antioxidant compounds.

### 2.5. Chromatographic Analysis of Polyphenolic Compounds

The chromatographic analysis of macerates and SFE extracts from yerba mate and yellow lupine was performed using HPLC-MS/MS for the content of selected biologically active compounds in order to compare and extend the results obtained by spectrophotometric analysis. In the qualitative and quantitative analysis, the monitoring reaction (MRM) mode was used. Table 2 shows the results of analysis of yerba mate and yellow lupine extracts obtained by SFE (t = 120 min, co-solvent flow = 0.4 mL/min) and maceration using 96% EtOH and 96% MeOH as co-solvents. The results of the determination of the extracts from both plants confirm the presence of key phenolic compounds. In the case of yerba mate, ten flavonoids and seven phenolic acids in SFE with 96% EtOH and nine flavonoids and eight phenolic acids in extracts with 96% MeOH were identified and determined. In both cases, chlorogenic acid was the dominant compound. While for yellow lupine nine flavonoids and six phenolic acids were identified in SFE extracts with 96% EtOH and 96% MeOH. In general, maceration extracts were characterized by a significantly lower content of polyphenolic compounds as compared to SFE extracts as indicated by the total value of phenolic compounds in the extracts. Only in case of yellow lupine, the total amount of polyphenols was higher for maceration with 96% MeOH than, than for SFE with 96% MeOH. Furthermore, in the case of yerba mate extracts from maceration, the dominant compound was the rutin, whose value in both cases was higher compared to the extracts from SFE (Table 2). Moreover, in contrast to earlier spectrophotometric determinations which showed 96% EtOH as much more efficient in extraction of polyphenolic compounds from yerba mate, the results of HPLC-MS/MS do not give such unambiguous results. The high level of flavonoids determined by HPLC-MS/MS, as well as phenolic acids is extremely interesting in comparison to results from spectrophotometric determinations, where in each of the extraction variants used, much less of these compounds were obtained than in extracts of yerba mate, which may suggest that previously used spectrophotometric methods are definitely less sensitive and selective.

### 2.6. Effect of Enzymatic Hydrolysis

**Figure 2:**
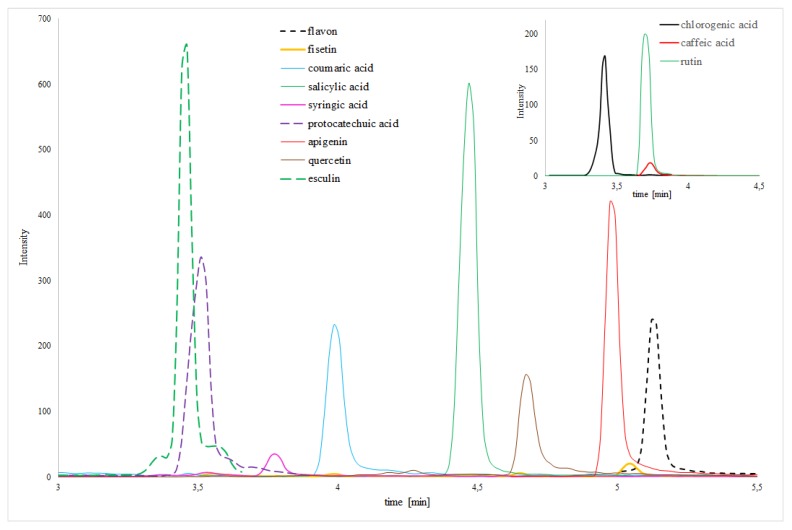
HPLC-MS/MS chromatograms of selected main compounds from yerba mate EA-SFE extract.

In the present study, SFE extracts obtained from enzyme-digested materials from yerba mate and yellow lupine were compared with the extracts obtained by maceration and SFE. For enzymatic hydrolysis, Kemzyme^®^ was used, according to the report by Mushtaq et al. [10]. As it may be seen in Table 2, analysis of the total polyphenolic compounds and the sum of individually determined phenolic acids and flavonoids showed that a proposed method of extraction using of commercial enzyme formulation is particularly effective in isolation of these substances (Figure 2).

For both plant materials, the content of tested compounds was more than three times higher for the protocol using SFE and enzymatic hydrolysis than for SFE alone.

The content of polyphenolic compounds varies notably depending on the extraction method and solvent used. In the case of yerba mate, the extraction method used allowed to significantly increase the content of quercetin, caffeic acid, chlorogenic acid, protocatechuic acid, as well as 4-hydroxy-benzoic acid, in comparison with both maceration and SFE extracts. The enormous increase in rutin and chlorogenic acid was observed for yellow lupine (Table 2).

**Figure 3:**
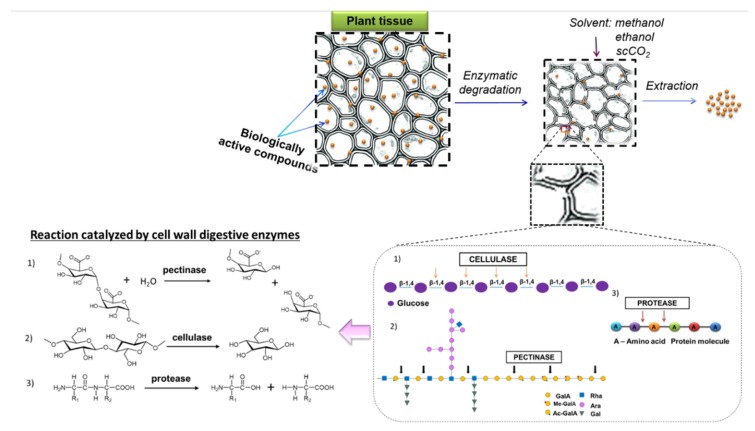
Scheme of enzymatic hydrolysis of plant cells prior solvent extraction.

The obtained results clearly indicate that applied so far methods of sample preparation and extraction of bioactive compounds from different plant materials are not satisfying. Conventional sample preparation techniques allow the isolation of only a small pool of compounds found in such a complicated matrix as particles of plant material. The developed protocol with mixture of different cell wall degrading enzymes is an efficient alternative for conventional techniques (Figure 3).

**Figure 4:**
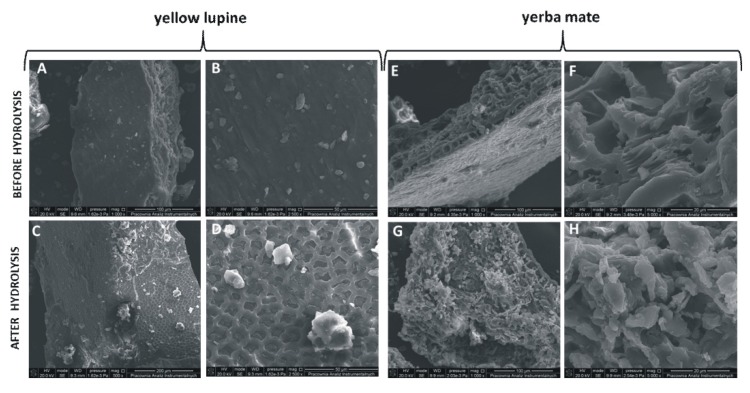
Comparison of the ultrastructure of yellow lupine plant material (**A**–**D**) and yerba mate plant material (**E**–**H**) before and after hydrolysis with Kemzyme^®^.

To confirm that enzymatic hydrolysis facilitates the isolation of polyphenolic compounds from plant material particles the scanning electron microscopy analysis (SEM) was used. This analysis allows verification the changes in the surface structure of yellow lupine and yerba mate tissues before and after enzymatic hydrolysis (Figure 4). It is obvious from Figure 4C,D,G,H that after the enzymatic hydrolysis, the plant materials are wrinkled and highly porous in comparison with control where is observed the smooth surface of plant particles (Figure 4A,B,E,F).

Overall, visible morphological changes indicate that the use of enzymatic hydrolysis before the extraction causes an increase in surface area which improves mass transfer rate and CO_2_ and co-solvent distribution and finally results in higher liberation efficiency of polyphenolic compounds [13,18].

### 2.7. Antibacterial Activity

Due to the fact that bioactive compounds carry out a variety of biological functions in plants, the obtained extracts may have different properties. For this reason, the antibacterial activity of the obtained extracts has been checked with selected bacterial strains of clinical and environmental significance. The minimum inhibitory concentration was determined for the extracts of yerba mate, and yellow lupine obtained by EA-SFE for 9 bacterial strains. The obtained results are summarized in Table 3.

Our studies showed that the obtained extracts effectively inhibited the growth of bacterial strains used. Extracts from the yerba mate shown the highest effectiveness against *S. aureus, A. baumannii* and *P. aeruginosa* an inhibition of growth of these strains was observed at 50 μg/mL. The weakest inhibition was noted for *E. coli, K. pneumoniae* and *S. infantis*, where the MIC value was 200 μg/mL. The yerba mate extract inhibited bacterial growth from of *B. subtilis, E. aerogenes* and *E. faecalis* at 100 μg/mL. Analysis of the antibacterial properties of yerba extracts obtained by various techniques carried out by Vieitez et al. [22] indicates that the extract obtained with 75% ethanol is characterized by the highest activity against *E. coli* and *S. aureus* with MIC values 570 and 280 μg/mL, respectively. The MIC value for the extract obtained with pure scCO_2_ was much higher and was above 3 mg/mL [22]. Presented results indicate that EA-SFE extract from yerba mate is much better inhibitor than extracts obtained with other techniques because it contains higher levels of different bioactive compounds. It comes from both their more efficient release from hydrolyzed plant material and combination of safe solvents that allow isolation a wider range of compounds.

The most effective inhibition of the growth of the bacterial strain in the case of yellow lupine extract occurred against *B. subtilis* for which the MIC was only 6.25 μg/mL. Among of the bacterial strains tested, *K. pneumoniae* and *S. infantis* were the most resistant to extracts of yellow lupine with MICs of 200 μg/mL. The presented MIC values are much lower than the MIC values for yellow lupine extract obtained by SFE with 96% EtOH as a co-solvent [5].

This clearly suggests that enzymatic hydrolysis before extraction is crucial for the amount of biologically active compounds released and EA-SFE is a particularly convenient technique for obtaining functional product that may in the future be used in the pharmaceutical, cosmetic or food industry. In both extracts there are high levels of bioactive compounds responsible for antibacterial properties such as chlorogenic acid and rutin. An inhibitory effect of chlorogenic acid on *Staphylococcus aureus*, *Trichosporon beigelii*, *Malassezia furfur*, *Shigella dysenteriae*, *Escherichia coli* and *Salmonella Typhimurium* was confirmed. In *S. aureus* cells treated with chlorogenic acid, a decrease in the level of intracellular ATP and release of cell constituents was observed. Studies indicate also reduction in intracellular pH and hyperpolarization of cell membrane [23]. Rutin exhibits antibacterial effects against *Streptococcus pyogenes, Enterococcus faecalis, Bacillus cereus, Pseudomonas aeruginosa, Klebsiella pneumoniae* and *Escherichia coli* [24]. Moreover, a synergistic effect of rutin with other flavonoids such as kaempherol, myricetin, fisetin or quercetin was reported [25]. In the obtained yellow lupine and yerba mate extracts, especially high antibacterial activity can be the result of synergistic effect of different flavonoids and phenolic acids.

## 3. Materials and Methods

### 3.1. Plant Materials and Extracting Solvent

Yerba mate was obtained from the company Lauro Raatz S.A. Yerba Mate Pajarito (Itapua, Paraguay). Seeds of yellow lupine were obtained from the Polish company Plantico (Waganiec, Poland). The plant materials were ground to fine powder using laboratory mill to homogenize and increase the specific surface area of the sample, then packed in paper bags and stored at room temperature in dark. The average particle diameter was less than 1 mm. In the next step, the obtained plant materials were extracted with using supercritical fluid extraction with co-solvent (SFE) and maceration for comparison purposes. Four different solvents were used for the extraction and all determinations, i.e., 70% and 96% (*v/v*) ethanol and methanol (Sigma Aldrich, Steinheim, Germany). Purity of CO_2_ was 99.99%.

### 3.2. Extraction Procedure

#### 3.2.1. Maceration

Four maceration variants were made for each material tested using four different solvents: I—ethanol 96%, II—ethanol 70%, III—methanol 96%, IV—methanol 70%. In order to prepare macerates for each variant, 2 g of the ground dried plant material was soaked in 20 mL of extractant for 24 h at 50 °C in the dark. After this time extracts were centrifuged (4000× *g*) (5810R Centrifuge, Eppendorf^TM^, Hamburg, Germany), then the clear supernatant was collected and stored at 4 °C until analysis.

#### 3.2.2. Supercritical Fluid Extraction with Co-Solvent (SFE)

The extraction process was carried out using a supercritical fluid extractor from MV-10 ASFE Systems (Waters Corporation, Milford, MA, USA) with compatible 5 mL extraction cells. The extraction equipment also includes a fluid delivery module (CO_2_ pump, co-solvent pump, co-solvent select valve), ThermoCube chiller, extraction oven, automatic back pressure regulator (ABPR), heat exchanger, fraction collection module and collection vessels. The extraction process was carried out in static and dynamic time. Ground samples (2 g) were placed in the extraction cell and completed with glass beads. In the course of the extraction process, the effect of the type of co-solvent, time of extraction and co-solvent flow rate on the effectiveness of isolation of biologically active compounds was examined.

##### Selection of Type and Flow Rate of Co-Solvent and Time of Extraction

In order to select the type of co-solvent, four variants of SFE were performed for each plant material tested using four different co-solvents, i.e., I—ethanol 96%, II—ethanol 70%, III—methanol 96%, IV—methanol 70%. The parameters of each of the four variants of the extraction process were: temperature—50 °C, pressure—300 bar, time—40 min (static time—30 min; dynamic time—10 min), the flow of scCO_2_—4 mL/min (0.936 g/min/g of feedstock) and co-solvent—1 mL/min. (0.395 g/min/g of feedstock) The extracts thus obtained were centrifuged and stored at 4 °C to carry out further analyses. In order to select the co-solvent flow rate, extraction was carried out with the reduced of co-solvent flow. The parameters of the extraction process were: temperature—50 °C, pressure—300 bar, extraction time—120 min (static—30 min, dynamic—90 min), CO_2_ flow—4 mL/min (0.936 g/min/g of feedstock), co-solvent flow—0.4 mL/min (0.169 g/min/g of feedstock).

In order to estimate the effect of time on the efficiency of the extraction process, three extraction variants were made with different dynamic time and constant static time of 30 min for each tested material: I—dynamic time: 15 min (total time—45 min), II—dynamic time: 30 min (total time—60 min), III—dynamic time: 90 min (total time—120 min). The others extraction conditions have not changed. The 96% ethanol was used as co-solvent.

### 3.3. Enzymatic Hydrolysis

The enzymatic hydrolysis of the aerial parts of yerba mate and seeds of yellow lupine with using the enzyme formulation Kemzyme^®^ was carried out according to the procedure optimized by Mushtaq et al. [10] with some modifications. Preferential cleavage and major active unit of enzyme formulation are shown in Table 4. Briefly, accurately weighted 2.0 g of ground material was mixed with the appropriate concentration of Kemzyme^®^ (2.9%) in 5 mL phosphate buffer (0.02 M, pH = 5.8) and incubated in 45 °C for 90 min. Afterwards, the formulation acquired was deactivated (in 90 °C for 5 min), degassed in ultrasonic bath (for 15 min) and dried in 50 °C. Then, the enzymatic treated and dried plant material underwent the supercritical fluid extraction with 96% EtOH as co-solvent, in properly selected conditions.

### 3.4. Determination of Dry Matter Content (DM)

In order to determine the yield of the extraction, dry masses of extracts were obtained by evaporation of 1 mL of each of the obtained extracts. The procedure was carried out in triplicate. Obtained dry matter (DM) content was expressed in % and calculated from the equation below:

(1) $$Y_{\mathrm{extract}}\left(\%\right)=\left(\frac{m_{\mathrm{extract}}}{m_{\mathrm{feed}}}\right)\times 100$$

where: Y_extract_ is extraction yield expressed in %, m_extract_ is the dry extract mass (g) and m_feed_ is the feed mass (g).

### 3.5. Determination of Total Phenolics Content (TPC)

The total phenolic content in each of extracts was determined using Folin–Ciocalteu (FC) method with analytical grade gallic acid as the standard by method previously described by Singleton et al. [26] with modifications. In brief, 100 µL of extract was added to 1.5 mL of deionized water and 100 µL Folin–Ciocalteu phenol reagent. The whole was mixed and incubated in the dark for 8 min, and then 300 μL of 20% sodium carbonate was added. After 30 min at 20 °C in the dark, the absorbance was measured at λ = 765 nm. The measurement was performed using NanoDrop 2000 spectrophotometer (Thermo Scientific, Waltham, MA, USA). The results were expressed as gallic acid equivalents (GAE) mg/g of dry matter extract.

### 3.6. Determination of Total Flavonoids Content (TFC)

Total flavonoid content in each extract was measured colorimetrically using the aluminium chloride (Sigma Aldrich, Steinheim, Germany) according to Rouphael et al. [27] with modifications. Briefly, 500 μL of each extract was mixed with the same volume of 2% AlCl_3_ and then diluted with solvent to 2 mL. After that, the whole was mixed and incubated 40 min at ambient temperature. The absorbance was measured against prepared reagent blank at λ = 415 nm using a NanoDrop 2000 spectrophotometer (Thermo Scientific, Waltham, MA, USA). The results were expressed as mg rutin equivalents (RE) using a calibration curve from rutin (Sigma Aldrich, Steinheim, Germany) solutions in 70% and 96% methanol and ethanol.

### 3.7. Determination of Antioxidant Properties Using the DPPH Radical Scavenging Method

The antioxidant properties of the extracts were determined using the 2,2-diphenyl-1-picrylhydrazyl (DPPH) radical scavenging method based on the procedure described by Espin et al. [28] with modifications. Tested extracts (300 μL) were added to DPPH solution (1.2 mL) and incubated on a shaker for 30 min in the dark at room temperature. After this time, the absorbance of the samples was measured at wavelength λ = 517 nm. The measurement was performed using the NanoDrop 2000 spectrophotometer. The results of the determinations were expressed as 6-hydroxy-2,5,7,8-tetramethylchroman-2-carboxylic acid equivalents (Trolox, TEAC, Sigma Aldrich, Steinheim, Germany) per gram of dry weight of the extract using a calibration curve from trolox solutions in 70% and 96% methanol and ethanol.

### 3.8. HPLC-MS/MS Analysis of Polyphenolic Compounds

The qualitative and quantitative analysis of plant extracts was carried out using an LC-MS 8050 chromatography system (Shimadzu, Kyoto, Japan) composed of a binary solvent delivery system (LC-30 AD), controller (CBM 20A), an autosampler (SIL-30A), and a column thermostat (CTO-20AC). The MS/MS analysis was performed in positive and negative mode on triple quadrupole equipped with an electrospray ionization (ESI) source. The optimal parameters of ESI-MS were as follows: interface temperature of 300 °C, DL temperature 250 °C, nebulizing gas flow 3 L/min, heating gas flow 10 L/min, and temperature of drying gas 400 °C. The biological active compounds were monitored in the scheduled multiple reaction monitoring (MRM) mode. The total dwell time was 0.7 s. Yellow lupine was separated using a Kinetex F5 column (100 × 2.1 mm, 2.6 µm, Phenomenex, Torrance, CA, USA), flow rate of 0.3 mL/min, injection volume of 10 µL, separation temperature of 25 °C, and the mobile phase consisting of acetonitrile and 0.1% HCOOC in water (gradient 0–7 min, 0–80% acetonitrile). In case of yerba mate extract used Kinetex C18 column (100 × 4.6 mm, 2.6 µm). The flow rate of the mobile phase was 0.3 mL/min, an injection volume was 10 μL, and separation temperature was 25 °C. 60% methanol and 40% 0.1% HCOOH in water were used as a mobile phase.

#### Preparation of Standard Solutions

The stock standard solutions of analytes at concentration 500 μg/mL were prepared in methanol. Afterwards, the stock standard solution was diluted in methanol. Calibration solutions in range 0.00005–10 μg/mL were prepared by dilution of the standard solution. Each point was the average of three injections with the injection volume of 10 μL. Integrated peak areas of the selected quantification MRM transitions were used to build the standard curves. In addition, the limit of detection (LOD) and the limit of quantification (LOQ) by dilution of working solutions under the HPLC-MS/MS conditions were determined for each standard. The LOD and LOQ are signal to noise ratio, three (S/N = 3) and ten (S/N = 10) times to noise level, respectively. Quantitative data were calculated from the calibration curves. Coefficient of correlation (R^2^), limit of detection (LOD) and limit of quantification (LOQ) are shown in Table 5. The prepared solutions were analyzed in positive and negative ionization mode for HPLC-ESI-MS/MS.

### 3.9. Scanning Electron Microscopy (SEM) Analysis

A scanning electron microscope/focused ion beam instrument (SEM/FIB) Quanta 3D FEG (FEI, Hillsboro, OR, USA) was applied to determine the surface structure of control and enzymatically hydrolyzed yellow lupine and yerba mate samples.

### 3.10. Determination of Antimicrobial Activity

To evaluate antibacterial properties of yerba mate and yellow lupine minimal inhibitory concentrations (MICs) were determined. The antibacterial activity of plant extracts was tested against *Staphylococcus aureus* ATCC25923, *Escherichia coli* ATCC25922, *B. subtillis* ATCC19659, *E. faecalis* ATCC14506, *A. baumannii* ATCCBAA–1605, *Pseudomonas aeruginosa* ATCC10145 and *Klebsiella pneumoniae* ATCC700603, *Salmonella infantis* (from the collection of the Collegium Medicum of Nicolaus Copernicus University, Torun, Poland). The minimal inhibitory concentration tests were performed according to Clinical and Laboratory Standards Institute (CLSI) guidelines. Bacterial strains were cultured on Mueller-Hinton broth and inoculated to the wells of a 96-microwell plate so final concentration of bacteria in each well was around 1 × 10^6^ CFU/mL. The dry extracts were dissolved in DMSO. The positive and negative controls were also performed. Cultures were grown for 18 h at 35 °C. Bacterial cells viability was read at 600 nm (Multiscan, ThermoFisher Scientific, Waltham, MA, USA). The MIC value corresponded to the dry extract concentration that inhibited bacterial growth.

## 4. Conclusions

The efficiency of extraction of polyphenolic compounds from yerba mate and yellow lupine depended on extraction time, type of solvent and co-solvent, and extraction method. The duration the extraction process significantly influences the final amount of extracted biologically active compounds. The co-solvent flow in SFE does not significantly change the final amount of biologically active compounds in the extract, so the process can be carried out with a reduced amount of the co-solvent used. HPLC-MS/MS analysis showed that the spectrophotometric methods used are less selective in relation to chromatographic methods. However, in particular, our study confirms that the enzyme-assisted supercritical fluid extraction (EA-SFE) approach is a promising method for isolation of bioactive compounds from different plant matrices. The results revealed that EA-SFE is more efficient in the extraction of polyphenolic compounds than maceration and SFE. Moreover, obtained extracts are characterized by high antibacterial and antioxidant activity which indicates a great practical application in different branches of industry. The developed extraction method is an eco-friendly green method, because it limits the consumption of toxic solvents and the energy necessary to isolate biologically active compounds.

## Figures and Tables

**Table 1:** Selection of the extraction method and solvent. Yields (%), total phenolic content (mg GAE/g DM), total flavonoid content (mg RE/g DM) and antioxidant activity obtained by the DPPH method (µmol TEAC/g DM) of the extracts obtained by different extraction methods and type of solvent as well as of the extracts obtained by SFE with different flow rate of co-solvent (96% EtOH) for different plants (T—50 °C, p—300 bar, time—120 min, flow rate CO_2_—4 mL/min).

**Yields**								
**Plant**	**Maceration MeOH 70%**	**SFE MeOH 70%**	**Maceration EtOH 70%**	**SFE EtOH 70%**	**Maceration MeOH 96%**	**SFE MeOH 96%**	**Maceration EtOH 96%**	**SFE EtOH 96%**
**Yerba mate**	17.50 ± 0.10	1.50 ± 0.09	16.20 ± 0.20	1.45 ± 0.05	17.90 ± 0.31	3.12 ± 0.03	17.90 ± 0.24	3.45 ± 0.03
**Yellow lupine**	10.09 ± 0.17	1.00 ± 0.11	9.72 ± 0.01	2.31 ± 0.09	13.30 ± 0.57	2.04 ± 0.01	10.55 ± 0.07	4.15 ± 0.01
**Total Phenolic Content**								
**Plant**	**Maceration MeOH 70%**	**SFE MeOH 70%**	**Maceration EtOH 70%**	**SFE EtOH 70%**	**Maceration MeOH 96%**	**SFE MeOH 96%**	**Maceration EtOH 96%**	**SFE EtOH 96%**
**Yerba mate**	65.74 ± 1.30	15.22 ± 0.97	80.33 ± 2.01	28.46 ± 1.94	92.19 ± 1.83	20.78 ± 3.01	135.48 ± 1.09	51.95 ± 2.06
**Yelllow lupine**	23.79 ± 0.97	11.38 ± 1.01	2.01 ± 1.71	11.08 ± 0.37	4.40 ± 1.50	20.49 ± 2.11	12.10 ± 0.57	15.00 ± 5.31
**Total Flavonoid Content**								
**Plant**	**Maceration MeOH 70%**	**SFE MeOH 70%**	**Maceration EtOH 70%**	**SFE EtOH 70%**	**Maceration MeOH 96%**	**SFE MeOH 96%**	**Maceration EtOH 96%**	**SFE EtOH 96%**
**Yerba mate**	9.04 ± 0.20	7.80 ± 0.50	9.17 ± 0.89	6.32 ± 0.44	5.22 ± 0.37	11.73 ± 1.01	21.39 ± 0.40	15.77 ± 0.77
**Yellow lupine**	-	-	1.01 ± 0.33	-	0.81 ± 0.09	-	5.55 ± 0.57	-
**Antioxidant Activity**								
**Plant**	**Maceration MeOH 70%**	**SFE MeOH 70%**	**Maceration EtOH 70%**	**SFE EtOH 70%**	**Maceration MeOH 96%**	**SFE MeOH 96%**	**Maceration EtOH 96%**	**SFE EtOH 96%**
**Yerba mate**	6.06 ± 0.22	12.47 ± 0.55	6.96 ± 0.35	14.76 ± 0.84	7.02 ± 0.37	0.72 ± 0.08	7.26 ± 0.39	28.37 ± 1.05
**Yellow lupine**	1.21 ± 0.19	4.50 ± 0.59	0.80 ± 0.12	3.69 ± 0.47	0.84 ± 0.29	5.41 ± 0.45	6.43 ± 0.78	10.75 ± 1.22
**Selection of the Co-Solvent Flow Rate**								
	**Yields (%)**		**TPC (mg GAE/g DM)**		**TFC (mg RE/g DM)**		**DPPH (µmol TEAC/g DM)**	
**Plant**	**1 mL/min EtOH 96%**	**0.4 mL/min EtOH 96%**	**1 mL/min EtOH 96%**	**0.4 mL/min EtOH 96%**	**1 mL/min EtOH 96%**	**0.4 mL/min EtOH 96%**	**1 mL/min EtOH 96%**	**0.4 mL/min EtOH 96%**
**Yerba mate**	6.42 ± 0.02	7.50 ± 0.50	90.65 ± 5.25	83.12 ± 1.23	32.05 ± 4.32	33.15 ± 2.51	122.14 ± 9.51	100.92 ± 2.05
**Yellow lupine**	9.60 ± 0.05	10.44 ± 0.92	53.45 ± 0.56	52.15 ± 1.01	10.04 ± 0.80	9.33 ± 4.44	31.34 ± 1.59	23.80 ± 0.50

All results were expressed as mean ± standard deviation (n = 3).

**Table 2:** Results of HPLC-MS/MS analysis of yerba mate and yellow lupine extracts.

		Yerba Mate						Yellow Lupine			
		Maceration MeOH	Maceration EtOH	SFE MeOH	SFE EtOH	EA-SFE EtOH	Maceration MeOH	Maceration EtOH	SFE MeOH	SFE EtOH	EA-SFE EtOH
**Compound**	**MRM**		**Concentration (µg/g)**								
**Flavonoids**											
**Flavone**	223-121	ND	0.005 ± 0.001	0.005 ± 0.001	0.02 ± 0.00	0.04 ± 0.01	0.004 ± 0.001	0.002 ± 0.001	0.07 ± 0.04	ND	0.06 ± 0.00
**Fisetin**	285-121	ND	ND	ND	ND	0.07 ± 0.02	0.47 ± 0.04	0.79 ± 0.13	ND	ND	0.21 ± 0.02
**Apigenin**	269-117	ND	ND	0.18 ± 0.01	0.26 ± 0.15	0.70 ± 0.03	17.85 ± 0.62	12.81 ± 0.31	1.55 ± 0.07	3.00 ± 0.28	18.82 ± 1.32
**Rutin**	609-300	66.13 ± 16.85	66.94 ± 6.03	58.09 ± 4.69	31.43 ± 2.43	67.98 ± 9.15	14.96 ± 1.63	10.94 ± 1.24	30.64 ± 0.23	54.24 ± 1.01	113.53 ± 3.06
**Quercetin**	301-227	7.45 ± 0.67	8.92 ± 1.43	4.09 ± 0.21	5.60 ± 2.01	20.38 ± 2.53	1.63 ± 0.27	3.06 ± 0.37	0.42 ± 0.09	1.05 ± 0.33	18.31 ± 2.16
**Naringin**	579-271	ND	ND	ND	0.0004 ± 0.0000	ND	2.88 ± 0.25	3.58 ± 0.13	0.13 ± 0.00	0.14 ± 0.01	0.01 ± 0.00
**Naringenin**	271-119	ND	ND	0.01 ± 0.00	0.01 ± 0.00	0.08 ± 0.00	0.50 ± 0.03	0.49 ± 0.05	0.16 ± 0.01	0.16 ± 0.02	0.72 ± 0.04
**Esculin**	339-177	0.11 ± 0.03	0.22 ± 0.05	0.99 ± 0.14	0.99 ± 0.37	1.11 ± 0.19	0.10 ± 0.06	0.08 ± 0.01	0.50 ± 0.03	0.23 ± 0.01	0.80 ± 0.09
**Esculetin**	177-89	ND	ND	0.71 ± 0.38	0.42 ± 0.18	5.25 ± 1.43	0.23 ± 0.03	0.28 ± 0.04	0.05 ± 0.03	0.13 ± 0.01	3.53 ± 0.65
**Biochanin A**	283-211	ND	ND	0.002 ± 0.001	0.007 ± 0.001	ND	0.006 ± 0.001	0.006 ± 0.001	ND	0.01 ± 0.00	0.01 ± 0.00
**Catechin**	289-123	ND	0.04 ± 0.01	0.03 ± 0.02	0.03 ± 0.00	0.75 ± 0.18	ND	ND	0.07 ± 0.02	0.04 ± 0.01	0.20 ± 0.02
⅀		73.70 ± 17.55	76.12 ± 7.52	64.13 ± 5.45	38.76 ± 5.14	96.35 ± 13.54	38.63 ± 2.93	32.02 ± 2.28	33.59 ± 0.52	59.00 ± 1.68	156.20 ± 7.36
**Phenolic acids**											
**Salicylic acid**	137-93	ND	ND	2.07 ± 0.77	1.37 ± 0.54	9.62 ± 1.05	0.84 ± 0.16	1.53 ± 0.09	0.63 ± 0.14	3.10 ± 0.03	4.77 ± 0.25
**Coumaric acid**	163-93	ND	ND	0.40 ± 0.01	ND	5.50 ± 0.84	2.21 ± 0.55	4.10 ± 0.26	0.94 ± 0.01	1.38 ± 0.13	6.89 ± 0.14
**Chlorogenic acid**	353-191	ND	ND	15.25 ± 4.97	39.40 ± 9.44	95.60 ± 11.56	9.50 ± 1.49	4.20 ± 0.79	18.88 ± 1.17	43.33 ± 3.03	130.10 ± 16.71
**Caffeic acid**	179-134	2.22 ± 0.93	3.18 ± 0.67	21.06 ± 2.90	13.82 ± 5.38	83.77 ± 9.67	1.43 ± 0.12	0.54 ± 0.10	0.61 ± 0.04	7.08 ± 0.34	13.43 ± 7.14
**Syringic acid**	197-95	ND	ND	4.72 ± 1.67	4.05 ± 1.98	5.66 ± 1.25	0.85 ± 0.11	0.66 ± 0.44	ND	0.62 ± 0.26	5.71 ± 0.02
**Protocatechuic acid**	153-108	ND	ND	1.94 ± 0.39	2.20 ± 0.88	13.22 ± 3.78	0.36 ± 0.10	0.22 ± 0.01	0.42 ± 0.10	ND	1.72 ± 0.11
**Sinapic acid**	223-149	ND	ND	0.59 ± 0.07	0.36 ±0.11	1.76 ± 0.46	0.41 ± 0.08	0.49 ± 0.10	0.13 ± 0.00	0.28 ± 0.03	0.74 ± 0.07
**4-Hydroxybenzoic acid**	137-65	ND	ND	0.27 ± 0.02	7.90 ± 0.00	34.31 ± 4.43	4.18 ± 0.88	6.89 ± 1.30	ND	ND	8.79 ± 4.30
⅀		2.22 ± 0.93	3.17 ± 0.67	46.30 ± 10.80	69.09 ± 18.33	249.42 ± 33.04	19.79 ± 3.49	18.63 ± 3.48	21.60 ± 1.46	55.78 ± 3.84	172.13 ± 28.74
⅀		75.92 ± 18.48	79.29 ± 8.19	110.43 ± 16.25	107.85 ± 23.47	345.77 ± 46.58	58.42 ± 6.42	50.65 ± 5.76	55.19 ± 1.98	114.78 ± 5.52	328.33 ± 36.10

ND—not detected.

**Table 3:** Minimal inhibitory concentration (MIC) for EA-SFE extract from yerba mate and yellow lupine.

Bacterial Species	Extract from Yerba Mate MIC [μg/mL]	Extract from Yellow Lupine MIC [μg/mL]
***S. aureus***	50	50
***E. coli***	200	100
***B. subtilils***	100	6.25
***E. aerogenes***	100	100
***E. faecalis***	100	100
***A. baumannii***	50	50
***P. aeruginosa***	50	25
***K. pneumoniae***	200	200
***S. infantis***	200	200

**Table 4:** Preferential enzymes and major active unit of enzyme formulation used.

Enzyme	E.C.	Major Units	Minimal Guaranteed Enzyme Activity[Units/g]
**Kemzyme^®^ Plus Concentrate dry**, (Kemin, Germany)	3.2.1.6	Endo-1,3 (4)-β-glucanase (β-glucanase) produced by *Aspergillus aculeatus* (CBS 589.94)	23,500
	3.2.1.4	Endo-1,4-β-glucanase (cellulase) produced by *Trichoderma longibrachiatum* (CBS 592.94)	180,000
	3.2.1.1	α-Amylase produced by *Bacillus amyloliquefaciens* (DSM 9553)	4000
	3.4.24.28	Bacillolysine (protease) produced by *Bacillus amyloliquefaciens* (DSM 9554)	17,000
	3.2.1.8	Endo-1,4-β-xylanase (xylanase) *Trichoderma viride* (NIBH FERM BP 4842)	350,000

**Table 5:** Validation of the method in HPLC-MS/MS.

Compound	Linear Regression Data			
	R^2^	LOD [ng/mL]	LOQ [ng/mL]	Range [ng/mL]
**Flavonoids**				
**Flavone**	0.9993	0.01	0.033	0.05–100
**Fisetin**	0.9998	0.1	0.33	0.5–100
**Apigenin**	0.9988	0.1	0.33	0.5–100
**Rutin**	0.9999	0.01	0.033	0.05–100
**Quercetin**	0.9999	1	3.3	5–500
**Naringin**	1.0000	0.01	0.033	0.05–100
**Naringenin**	0.9999	0.01	0.033	0.05–100
**Esculin**	0.9997	0.01	0.033	0.05–100
**Esculetin**	0.9987	0.1	0.33	0.5–100
**Biochanin a**	0.9997	0.01	0.033	0.05–100
**Catechin**	0.9998	1	3.3	5–500
**Phenolic acids**				
**Salicylic acid**	0.9996	0.1	0.33	0.5–100
**Coumaric acid**	0.9987	0.5	1.65	5–400
**Chlorogenic acid**	0.9992	0.5	1.65	5–1000
**Caffeic acid**	0.9999	0.5	1.65	5–1000
**Syringic acid**	0.9978	50	165	500–10000
**Protocatechuic acid**	0.9995	1	3.3	5–1000
**Sinapic acid**	0.9992	1	3.3	5–1000
**4-Hydroxybenzoic acid**	0.9925	50	165	50–10000

LOD—limit of detection; LOQ—limit of quantification.
